# Characterization and regulation of wild‐type and mutant TASK‐1 two pore domain potassium channels indicated in pulmonary arterial hypertension

**DOI:** 10.1113/JP277275

**Published:** 2018-11-24

**Authors:** Kevin P. Cunningham, Robyn G. Holden, Pilar M. Escribano‐Subias, Angel Cogolludo, Emma L. Veale, Alistair Mathie

**Affiliations:** ^1^ Medway School of Pharmacy University of Kent and University of Greenwich Chatham Maritime Kent UK; ^2^ Department of Pharmacology and Toxicology School of Medicine University Complutense of Madrid Instituto de Investigación Sanitaria Gregorio Marañón (IiSGM) Madrid Spain; ^3^ Ciber Enfermedades Respiratorias (CIBERES) Madrid Spain; ^4^ Red de Investigación Cardiovascular Instituto de Salud Carlos III Madrid Spain

**Keywords:** Pulmonary arterial hypertension, KCNK3 (TASK‐1) potassium channel, riociguat

## Abstract

**Key points:**

- The TASK‐1 channel gene (KCNK3) has been identified as a possible disease‐causing gene in heritable pulmonary arterial hypertension (PAH).
- In the present study, we show that novel mutated TASK‐1 channels, seen in PAH patients, have a substantially reduced current compared to wild‐type TASK‐1 channels.
- These mutated TASK‐1 channels are located at the plasma membrane to the same degree as wild‐type TASK‐1 channels.
- ONO‐RS‐082 and alkaline pH 8.4 both activate TASK‐1 channels but do not recover current through mutant TASK‐1 channels.
- We show that the guanylate cyclase activator, riociguat, a novel treatment for PAH, enhances current through TASK‐1 channels but does not recover current through mutant TASK‐1 channels.

**Abstract:**

Pulmonary arterial hypertension (PAH) affects ∼15–50 people per million. KCNK3, the gene that encodes the two pore domain potassium channel TASK‐1 (K2P3.1), has been identified as a possible disease‐causing gene in heritable PAH. Recently, two new mutations have been identified in KCNK3 in PAH patients: G106R and L214R. The present study aimed to characterize the functional properties and regulation of wild‐type (WT) and mutated TASK‐1 channels and determine how these might contribute to PAH and its treatment. Currents through WT and mutated human TASK‐1 channels transiently expressed in tsA201 cells were measured using whole‐cell patch clamp electrophysiology. Localization of fluorescence‐tagged channels was visualized using confocal microscopy and quantified with in‐cell and on‐cell westerns. G106R or L214R mutated channels were located at the plasma membrane to the same degree as WT channels; however, their current was markedly reduced compared to WT TASK‐1 channels. Functional current through these mutated channels could not be restored using activators of WT TASK‐1 channels (pH 8.4, ONO‐RS‐082). The guanylate cyclase activator, riociguat, enhanced current through WT TASK‐1 channels; however, similar to the other activators investigated, riociguat did not have any effect on current through mutated TASK‐1 channels. Thus, novel mutations in TASK‐1 seen in PAH substantially alter the functional properties of these channels. Current through these channels could not be restored by activators of TASK‐1 channels. Riociguat enhancement of current through TASK‐1 channels could contribute to its therapeutic benefit in the treatment of PAH.

## Key points

- The TASK‐1 channel gene (KCNK3) has been identified as a possible disease‐causing gene in heritable pulmonary arterial hypertension (PAH).
- In the present study, we show that novel mutated TASK‐1 channels, seen in PAH patients, have a substantially reduced current compared to wild‐type TASK‐1 channels.
- These mutated TASK‐1 channels are located at the plasma membrane to the same degree as wild‐type TASK‐1 channels.
- ONO‐RS‐082 and alkaline pH 8.4 both activate TASK‐1 channels but do not recover current through mutant TASK‐1 channels.
- We show that the guanylate cyclase activator, riociguat, a novel treatment for PAH, enhances current through TASK‐1 channels but does not recover current through mutant TASK‐1 channels.

## Introduction

Pulmonary arterial hypertension (PAH, Group 1 pulmonary hypertension) (Simonneau *et al*. 2013, Galie *et al*. 2015) is a progressive and incurable disease that occurs when the pulmonary arteries undergo pathological remodelling, causing the blood vessels to vasoconstrict, with a consequent increase in pulmonary vascular resistance. Continued high pressure in the pulmonary artery leads to right ventricular heart failure and death if left untreated (Gaine & McLaughlin, 2017). Although PAH is a rare disease with an incidence of 15–50 people per million in the population (Humbert *et al*. 2014), the prognosis for patients is poor, with a 5 year survival rate of 34% (Tang *et al*. 2016). PAH can be either idiopathic (iPAH), heritable/familial (hPAH), or associated with other pathological conditions such as connective tissue diseases (e.g. scleroderma), human immunodeficiency virus (HIV), advanced liver disease, congenital heart disease and Schistosomiasis infection (Butrous 2015, Garg *et al*. 2017, Ghofrani *et al*. 2017). Despite being an incurable disease, there are now treatments and medication that can delay disease progression and increase life expectancy. Currently, there are three known pathways that contribute to cell proliferation and vasoconstriction in the pulmonary arteries: the prostacyclin, endothelin and nitric oxide pathways. Clinical treatments are aimed at appropriate up‐ or down‐regulation of each of these pathways to slow disease progression (Hill *et al*. 2016). Recently, riociguat (BAY 63–2521), a guanylate cyclase stimulator, acting downstream of nitric oxide through increased cGMP production, has been approved for use in PAH (Hill *et al*. 2016, Ghofrani *et al*. 2017). The therapeutic benefits of riociguat include improvement in pulmonary vascular haemodynamics and increased exercise ability in patients with PAH.

Advances in DNA sequencing and wide availability of whole exome sequencing has resulted in a large number of genetic defects being identified in both hPAH and iPAH sufferers, enhancing the molecular understanding of the pathogenic mechanism(s) underlying the disease. Both hPAH and iPAH have been linked to a defective copy of the bone morphogenic protein receptor type II (BRPM2) gene, which regulates vascular cell proliferation (Machado *et al*. 2015). Other, less frequent, mutations associated with PAH include ALK1, ENG, TBX4, EIF2AK4, SMAD, CAV1 and NOTCH3 (Tang *et al*. 2016). Additionally, a number of ion channels have been identified as risk factors for hPAH and iPAH. In particular, two potassium ion channels, Kv1.5 (KCNA5) and TASK‐1 (KCNK3, K_2P_3.1), which are implicated in hPAH and iPAH development, play a crucial role in regulating pulmonary vascular tone (Yuan *et al*. 1998, Boucherat *et al*. 2015, Hemnes & Humbert 2017, Olschewski *et al*. 2017). Down‐regulation, inhibition and mutations of these channels resulting in loss of channel function have been shown to contribute to cell proliferation, resistance to apoptosis and vasoconstriction in pulmonary arterial smooth muscle cells (PASMCs) (Remillard *et al*. 2007, Antigny *et al*. 2016). Furthermore, a reduction in TASK‐1 channel function has been observed in right ventricular cardiomyoctes prior to the development of right ventricular hypertrophy related to pulmonary hypertension (Lambert *et al*. 2018).

TASK‐1 channels belong to the two pore domain family of potassium ion channels (Enyedi & Czirjak 2010). Six heterozygous TASK‐1 mutations were first described in 2013, for both hPAH and iPAH patients (Ma *et al*. 2013). Characterization of these mutations using patch clamp electrophysiology showed that, when expressed homozygously, these mutations resulted in loss of channel function. However, the application of a phospholipase A2 inhibitor (ONO‐RS‐O82) was able to partially restore channel function for two of the homozygous mutant channels, thus opening a new avenue for PAH‐directed therapeutic development (Girerd *et al*. 2014). This report was followed by the identification of two further mutations, this time homozygous mutations, from a Spanish cohort of PAH patients, with an aggressive form of the disease (Navas *et al*. 2016).

In the present study, we characterize the functional properties and membrane localization of the two new homozygous mutations (G106R and L214R) identified by Navas *et al*. (2016). We show that current through these channels is considerably reduced compared to WT TASK‐1 channels and that functional current through these mutated channels cannot be restored using activators of WT TASK‐1 channels (pH 8.4, ONO‐RS‐082). In addition, we show for the first time that the guanylate cyclase activator, riociguat, enhances current through WT TASK‐1 channels. Similar to the other activators, however, riociguat does not have any effect on current through G106R or L214R mutated TASK‐1 channels. A preliminary account of some of these results has been reported previously (Cunningham *et al*. 2017).

## Methods

### Molecular biology

Human wild‐type (WT) TASK‐1 (KCNK9) cDNA was cloned into pcDNA3.1 vector (Invitrogen, Carlsbad, CA, USA) and was a kind gift from Helen Meadows (GlaxoSmithKline, Harlow, UK). WT TASK‐1 was cut out of pcDNA3.1 vector and cloned into the MCS of pAcGFP1‐N1 vector (Clontech‐Takara Bio Europe, Saint‐Germain‐en‐Laye, France) to create a fusion construct with the N‐terminus of AcGFP1. The terminating stop codon of TASK‐1 was removed by site‐directed mutagenesis and maintained in the same reading frame with the start codon of AcGFP1. Constructs were fully sequencing to ensure correct sequence incorporation (Eurofins MWG Operon, Huntsville, AL, USA).

### Mutations

Point mutations were introduced into both pcDNA3.1 and pAcGFP1‐N1 encoding TASK‐1 cDNA by site‐directed mutagenesis using a QuikChange site‐directed mutagenesis kit and procedure (Stratagene, La Jolla, CA, USA). A pair of complementary oligonucleotide primers (25–35 bases) incorporating the intended mutation (either G106R or L214R) were synthesized (Eurofins MWG Operon, Ebersberg, Germany). All constructs were fully sequenced to ensure correct mutation incorporation.

### Cell culture

tsA201 cells, which are modified human embryonic kidney 293 cells stably transfected with the SV40 large T antigen (ECACC; Sigma‐Aldrich, Gillingham, Dorset, UK), were grown in a monolayer tissue culture flask maintained in growth medium that was composed of 88% minimum essential media with Earle's salts, 2 mm l‐glutamine, 10% heat‐inactivated foetal bovine serum, 1% penicillin (10,000 units mL^−1^) and streptomycin (10 mg mL^−1^), and 1% nonessential amino acids (Sigma‐Aldrich; Pan Biotech, Aidenbach, Germany; Fisher Scientific, Pittsburgh, PA, USA). The cells were stored in an incubator at 37 °C with a humidified atmosphere of 95% O_2_ and 5% CO_2_. When the cells reached 80% confluency, they were were split and resuspended in a four‐well plate containing glass coverslips (diameter 13 mm) coated with poly‐d‐lysine (1 mg mL^–1^) at a concentration of 7 × 10^4^ cells in 0.5 mL of media, ready for transfection the next day.

### Transfection

For the electrophysiological experiments, cells were transiently transfected using a modified calcium‐phosphate protocol described by Chen & Okayama (1987). Next, 500 ng of pcDNA3.1 vector (Invitrogen) encoding human WT or mutant human TASK‐1 (KCNK3) (Genbank AF006823) and 500 ng of cDNA encoding for green fluorescent protein (GFP) was added into each well. The cells were incubated for 6–8 h at 37 °C in 95% O_2_ and 5% CO_2_. Following incubation, cells were washed twice with a 1 × PBS, and incubated in 0.5 mL of fresh growth media overnight. The cells were used for electrophysiological recordings the next day.

For confocal microscopy, cells were transfected using TurboFect transfection reagent (ThermoFisher, Loughborough, UK). Then, 1 μg of pAcGFP1‐N1 vector (Clontech‐Takara Bio Europe) encoding human WT or mutant human TASK‐1 was diluted in 100 μL of serum‐free cell media. To this, 1.5 μL of the Turbofect transfection reagent was added and incubated for 15–20 min. After incubation, this mixture was added to one well of the plate and incubated for 24–48 h at 37 °C in 95% O_2_ and 5% CO_2_, prior to membrane staining and/or fixation.

### Membrane staining

The plasma membranes of cells were stained using CellMask Deep Red (Thermofisher). A freshly prepared 1:1000 dilution of CellMask Deep Red plasma membrane stain (1×) was prepared in 1 × PBS. Transfected cells were then washed three times with 1 × PBS and submerged into 1 mL of prepared CellMask membrane stain for 5–10 min at 37 °C. The staining solution was removed by washing the coverslips twice in PBS, ready for fixation.

### Cell fixation

Prior to fixation cells were washed twice with room temperature 1 × PBS and then fixed in 1 mL of 2% paraformaldehyde (PFA) solution and incubated at 4 °C for 20 min. Following incubation, the cells were washed twice in 1 × PBS. For nuclei staining, 500 μL of freshly prepared Hoechst 33528 (1:500 dilution in PBS; Sigma‐Aldrich) was added to each well and incubated at 37 °C for up to 10 min. Cells were then washed twice with PBS and rinsed once with ddH_2_O to remove any salt crystals. Coverslips were mounted face down onto slides containing a drop of Vectashield anti‐fade mounting medium (Vector Laboratories, Peterborough UK).

### Confocal microscopy

Images were taken using a LSM 880 confocal microscope (Carl Zeiss, Oberkochen, Germany), placed upon an anti‐vibration table and processed using Zen Black software (Carl Zeiss). Cells were imaged using oil immersion under a Plan‐Apochromat 63×/1.4 oil DIC M27 objective (Carl Zeiss). The cells were excited with an argon laser at either 561, 488 or 405 nm for the CellMask Deep Red plasma membrane stain, pAcGFP‐channel and Hoechst 33528, respectively.

### Co‐localization analysis

Zen Black software was used to determine the degree of co‐localization. Regions of interest around cells with detectable green fluorescence were selected for analysis. Control images for pAcGFP‐only cells and Deep Red membrane stain‐only cells were taken with each set of images to ensure co‐localized quadrants were not set arbitrarily. Pearson's correlation coefficient (PCC) was used to represent the degree of co‐localization.

### In‐cell and on‐cell western

Cells were seeded onto a poly‐d‐lysine coated 96‐well plate at a density of 2 × 10^4^ cells/well (in 100 μL). The cultures were incubated for 24 h at 37 °C in 5% CO_2_ until a confluence of 80–95% was reached. Each well was transfected with 200 ng of DNA for HA‐tagged WT or mutated TASK1 and 0.4 μL of Turbofect and incubated for 24 h at 37 °C in 5% CO_2_. Cell culture media was removed and cells were immediately fixed with 40 μL of 2% PFA and incubated at room temperature for 20 min on the bench. Fixing solution was removed and cells washed.

For staining, cells were incubated 40 μL of a 1:500 dilution of 2 μg μL^–1^ monoclonal anti‐HA antibody (mouse) in blocking solution. A 1:1000 dilution IRDye 800CW (green) goat anti‐mouse secondary antibody and DRAQ5 (1:10,000; 5 mm) was added to each well. For whole cell staining (in‐cell), the membrane was permeablized with 40 μL of 0.1% Triton X‐100. Plates were scanned with detection in both the 700  and 800 nm channels using a Odyssey SA near‐infrared fluorescence imager (Li‐Cor, Lincoln, NE, USA). Integrated intensities were recorded and analysed. For each experiment, each condition was repeated in triplicate (three coverslips) and each experiment was repeated on three separate independent occasions.

### Whole‐cell patch clamp electrophysiology

Currents were recorded from tsA201 cells transiently transfected with the channel of interest using whole‐cell patch clamp in a voltage clamp configuration. A coverslip with transfected tsA201 cells was transferred into a recording chamber filled with an external solution composed of (in mm) 145 NaCl, 2.5 KCL, 3 MgCl_2_, 1 CaCl_2_ and 10 Hepes (pH to either 7.4 or 8.4, using NaOH), mounted under an inverted microscope (Diaphot; Nikon, Tokyo, Japan) with epifluorescence. External solution and modulatory compounds were superfused at a rate of 4–5 mL min^−1^. Complete exchange of the bath solution occurred within 100–120 s. Only cells that were transfected with GFP as evident from green fluorescence (excitation 395–440 nm; emission 470–600 nm) were selected for electrophysiological recordings. Patch pipettes were pulled from thin walled borosilicate glass (GC150TF; Harvard Apparatus, Edenbridge, UK) and had resistances of 3–6 MΩ when filled with pipette solution. The pipette solution contained (in mm) 150 KCL, 3 MgCl_2_, 5 EGTA and 10 Hepes (pH adjusted to 7.4 with KOH). Whole‐cell currents were recorded at a holding potential of –60 mV at 20–24 °C (room temperature). Cells were hyperpolarized to –80 mV for 100 ms and then subjected to a step to –40 mV for 500 ms, followed by a a step to –120 mV for 100 ms. These step changes were followed by a 500 ms voltage ramp to +20 mV and a step back to –80 mV for another 100 ms before returning to the holding potential of –60 mV. This protocol was composed of sweeps lasting 1.5 s, including sampling at the holding voltage and was repeated once every 5 s. Currents were recorded using an Axopatch 200 or 1D patch clamp amplifier (Molecular Devices, Sunnyvale, CA, USA) and analysed using pCLAMP, version 10.2 (Molecular Devices), Excel (Microsoft Corp., Redmond, WA, USA) and Prism, version 6 or 7 (GraphPad Software Inc., San Diego, CA, USA). For analysis of outward current, we measured the current amplitude during the step to −40 mV.

### Chemicals

ONO‐RS‐082 was purchased from Abcam (Cambridge, UK) and dissolved in dimethyl sulphoxide (DMSO) to create a 10 mm stock solution. This was diluted in external solution to the desired concentration just before use. Riociguat was purchased from MedChemExpress (Shanghai, China), diluted in DMSO to create a 10 mm stock. All other chemicals were purchased from Sigma‐Aldrich.

### Statistical analysis

Data are expressed as the mean ± 95% confidence intervals (CI) or the mean ± SEM, where *n* represents the number of individual cells, and ‘days’ represents the number of different recording days. On any given day, recordings were randomized to include both control and treatment recordings, for both electrophysiological and imaging experiments. Statistical analysis was performed using Prism, version 6 or 7 (GraphPad Software Inc.). Statistical analysis used one‐way ANOVA with a *post hoc* Dunnett's test for multiple comparison tests, as well as paired or unpaired Student's *t* tests. *P* < 0.05 was considered statistically significant. Where appropriate, the 95% CI for the difference in means is also given.

## Results

### Structural analysis of the human TASK‐1 mutations

**Figure 1:**
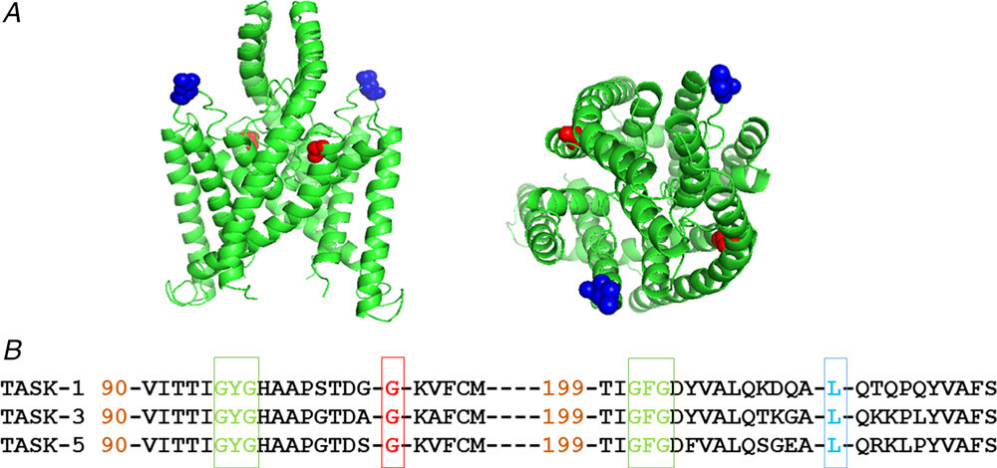
Homology model of TASK‐1 variants *A*, homology model of TASK channels based on TRAAK crystal structure (PDB ID: 3UM7; Brohawn *et al*. 2012) depicting the location of the two PAH TASK‐1 mutations: G106R and L214R. TASK‐1_G106 amino acids are shown in red and TASK‐1_L214 are shown in blue. Left: side view of the channel. Right: view from above the channel. *B*, amino acid sequence alignment of TASK‐1 with the two other members of the TASK subfamily: TASK‐3 and TASK‐5. Gaps are indicated by dashes and numbers indicate where sequence begins relative to the full length channel. The amino acids mutated in PAH patients, glycine (G) 106 and leucine (L) 214 are in red and blue, respectively. The selectivity filter regions are shown in green. [Color figure can be viewed at wileyonlinelibrary.com]

TASK‐1, which is encoded by the gene KCNK3, belongs to the two‐pore domain potassium (K2P) superfamily of potassium ion channels. The K2P family of ion channels is characterized by a unique molecular topology consisting of two α‐subunits that dimerize together to produce a single central pore, with each α‐subunit comprising two pore (P) loop forming domains and four transmembrane domains (Enyedi & Czirjak 2010). Two novel pathogenic missense TASK‐1 mutations were identified by Navas *et al*. (2016) from three Spanish patients with an aggressive form of iPAH and hPAH. These mutations c.614 T>G (L214R) and c.316 G>C G106R were identified on exon 2 of KCNK3 and were the first homozygous mutations to be reported in PAH (Navas *et al*. 2016). Using a generic TASK channel homology model, based on a crystal structure of the K2P channel TRAAK (Brohawn *et al*. 2012), the positions of the two mutations (on the two subunits in the TASK channel dimer) are indicated in Fig. 1 *A*. The glycine (G) 106 [mutated to an arginine (R)] is located extracellularly between the first pore domain and the second transmembrane domain, whereas the leucine (L) 214 (also mutated to R) is located extracellularly between the second pore domain and the fourth transmembrane domain. Alignment of TASK‐1 with other members of the acid‐sensitive TASK subfamily (TASK‐3 and TASK‐5) shows that these are highly conserved residues and are probably critical for channel function (Fig. 1 *B*).

### Characterization of current through G106R and L214R mutated human TASK‐1 channels

**Figure 2:**
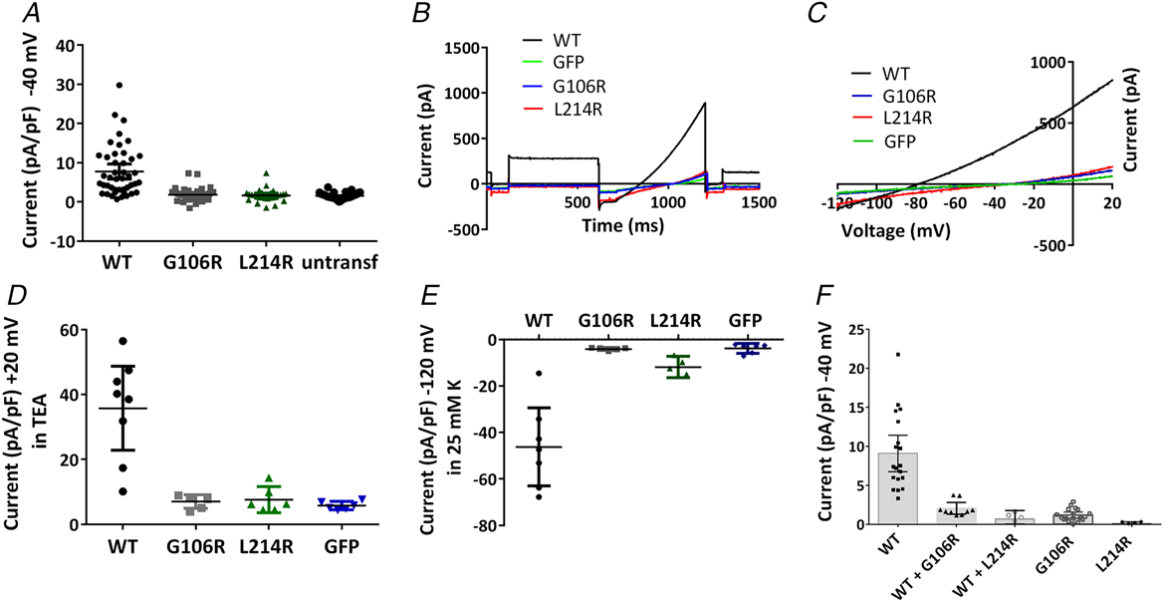
Electrophysiological profiling of currents through WT TASK‐1 and TASK‐1 mutant channels *A*, current density (pA pF^–1^) measured at –40 mV from individual cells transiently expressing WT TASK‐1, TASK‐1_G106R, TASK‐1_L214R channels or untransfected cells. Error bars represent the 95% CI. *B*, raw data trace from exemplar human TASK‐1, TASK‐1_G106R, TASK‐1_L214R channels and GFP alone transfected cells in 10 mm TEA using a step‐ramp voltage protocol as detailed in the Methods. *C*, current–voltage relationship for the cells in (*B*) evoked by ramp changes in voltage from –120 to –20 mV. *D*, current density (pA pF^–1^) measured at +20 mV in the presence of 10 mm TEA for cells expressing WT TASK‐1, TASK‐1_G106R, TASK‐1_L214R channels or GFP alone. *E*, current density (pA pF^–1^) measured at –120 mV in 25 mm K external for cells expressing WT TASK‐1, TASK‐1_G106R, TASK‐1_L214R channels or GFP alone. *F*, current density (pA pF^–1^) measured at –40 mV for cells expressing WT TASK‐1, TASK‐1_G106R, TASK‐1_L214R channels or co‐expression of WT TASK_1 and TASK‐1_G106R or WT TASK_1 and TASK‐1_L214R. [Color figure can be viewed at wileyonlinelibrary.com]

Previously identified mutations of TASK‐1 from PAH patients were found to cause loss of function of the channel at physiological pH when expressed transiently as homodimer mutants (Ma *et al*. 2013). Whole‐cell patch clamp techniques were utilized to determine functionality of the two novel homodimer TASK‐1 mutants, G106R and L214R. Whole‐cell recordings from tsA201 cells transiently transfected with human TASK‐1 cDNA gave whole cell currents of 7.8 pA pF^–1^ (*n* = 44 cells from 17 days, 95% CI = 5.8–9.7) (measured as current density at –40 mV; see Methods) (Fig. 2 *A*), using a physiological extracellular solution of 2.5 mm [K^+^]_o_. Ramp changes in holding potential from –120 to +20 mV demonstrate that the current is outwardly rectifying (Fig. 2 *B* and *C*) with a mean zero current potential of –78 mV (*n* = 44, 95% CI = –74 to –82) close to the equilibrium potential for potassium ions under these recording conditions. We have shown previously that TASK‐1 channels expressed in tsA201 cells are enhanced by alkaline pH, blocked by acidic pH and blocked by methanandamide (Aller *et al* 2005, Veale *et al* 2007).

By contrast, mutation of a small uncharged glycine (G) residue at position 106 to a large positively charged arginine (R) residue, resulted in a poorly functioning channel with significantly reduced current, when expressed alone as a homozygous mutant channel in tsA201 cells (Fig. 2 *A*–*C*). The average whole‐cell current measured at –40 mV was 1.8 pA pF^–1^ (*n* = 38 cells from 14 days, 95% CI = 1.3–2.4). Similarly, mutation of a small hydrophobic leucine (L) residue at position 214 to a large positively charged arginine (R) residue was found to have substantially reduced current when expressed alone as a homozygous mutant channel in tsA201 cells (Fig. 2 *A*–*C*). The average whole‐cell current measured at –40 mV was 1.6 pA pF^–1^ (*n* = 27 cells from 10 days, 95% CI = 1.0–2.3). The outward currents recorded from TASK‐1_G106R and TASK‐1_L214R channels were statistically significantly reduced (*P* < 0.05, one‐way ANOVA followed by a Dunnett's multiple comparisons test) from WT TASK‐1 and not significantly different from untransfected cells. Untransfected tsA201 cells had an average whole‐cell current measured at –40 mV of 1.5 pA pF^–1^ (*n* = 36 cells from 9 days, 95% CI = 1.2–1.8) (Fig. 2 *A*–*C*).

**Figure 3:**
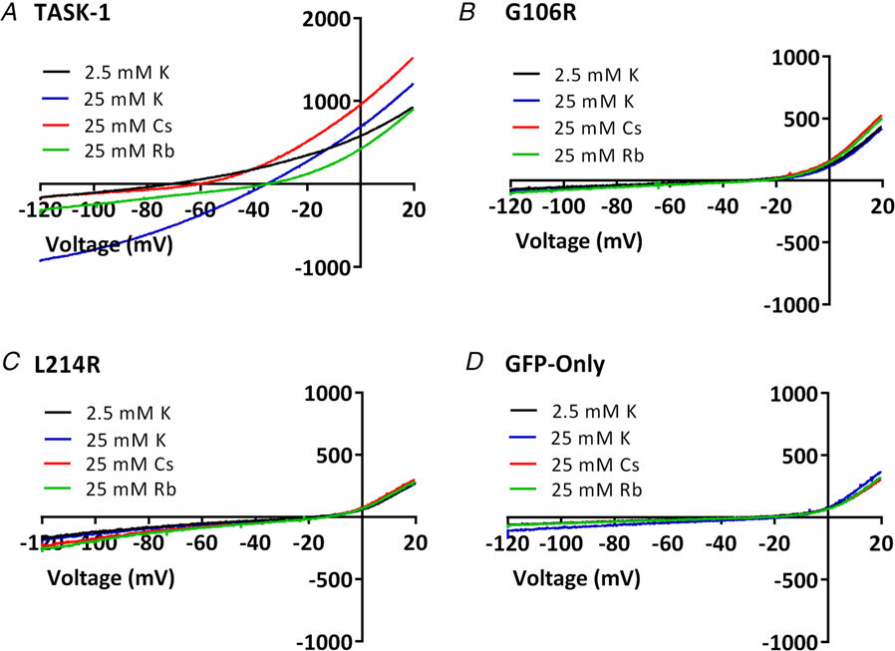
Ion permeability of TASK‐1 and mutated TASK‐1 channels *A*–*D*, current–voltage relationships from exemplar human TASK‐1, TASK‐1_G106R, TASK‐1_L214R channels and GFP alone transfected cells in 2.5 mm K, 25 mm K, 25 mm Rb or 25 mm Cs. [Color figure can be viewed at wileyonlinelibrary.com]

Outward currents at +20 mV were recorded in the presence of tetraethylammonium chloride (TEA) (10 mm) to minimize the contribution of currents through endogenous K_V_ channels in tsA201 cells. As for outward currents at –40 mV, currents through both mutated channels at +20 mV were significantly smaller than current through WT TASK‐1 channels and not significantly different from cells transfected with GFP alone (Fig. 2 *D*). Inward currents though WT and mutated TASK‐1 channels were measured at –120 mV, when the external solution was raised to 25 mm K to identify inward current through WT channels (Fig. 3). Again, inward currents through both mutated channels at –120 mV were significantly smaller than current through WT TASK‐1 channels and not significantly different from cells transfected with GFP alone (Fig. 2 *E*)

### Heterodimeric channels

Co‐expression of either of the mutated TASK‐1 channels with WT TASK‐1 channels to allow the formation of heterodimeric channels gave substantially and significantly reduced currents from WT TASK‐1 channels alone (*P* < 0.05, one‐way ANOVA) that were not significantly different from mutated channels alone (Fig. 2 *F*). This suggests that the mutated channels are dominant negative, when expressed heterologously with WT TASK‐1 channels.

### Ion selectivity of WT and mutated TASK‐1 channels

Ramp changes in voltage from –120 to +20 mV were applied to cells expressing WT TASK_1 channels, both of the mutant channels or GFP alone in normal K external solution (2.5 mm). The reversal potential of the current was obtained from these ramps and compared with that obtained with an external solution containing 25 mm K (Fig. 2). The external solution was then changed again to one where 25 mm of Rb or Cs replaced the 25 mm K. For WT TASK‐1 channels, clear shifts in *V*_rev_ were seen compared to recordings in 25 mm K (Fig. 3 *A* and Table 1). By contrast, no changes in current reversal potential were seen for either mutant channel in any condition and the reversal potentials seen were not significantly different from that seen for cells transfected with GFP alone (Fig. 3 *B–D* and Table 1).

**Table 1:** The effect of permeating ion on TASK‐1 channel current reversal potential

	2.5 mm K	25 mm K	25 mm Cs	25 mm Rb
TASK‐1	–73 ± 2 (*n *= 13)	–38 ± 1 (*n *= 10)	–64 ± 2 (*n *= 8)	–41 ± 3 (*n *= 7)
TASK‐1_G106R	–33 ± 8 (*n *= 5)	–28 ± 4 (*n *= 5)	–29 ± 5 (*n *= 5)	–25 ± 3 (*n *= 5)
TASK‐1_L214R	–19 ± 3 (*n *= 5)	–17 ± 2 (*n *= 5)	–18 ± 4 (*n *= 5)	–15 ± 3 (*n *= 5)
GFP alone	–32 ± 2 (*n *= 13)	–25 ± 3 (*n *= 6)	–31 ± 4 (*n *= 5)	–27 ± 3 (*n *= 5)

Reversal potentials (in mV) obtained for TASK‐1, TASK‐1_G106R, TASK‐1_L214R and GFP alone transfected cells when the external solution contained 2.5 mm K, 25 mm K, 25 mm Cs or 25 mm Rb.

### Cellular localization of fluorescence‐labelled TASK‐1 variants

To determine whether the reduced current levels recorded through TASK‐1_G106R and TASK‐1_L214R variants were a consequence of reduced trafficking to the plasma membrane, fluorescence‐tagged channels were used to examine cellular localization. GFP was fused to the C terminus of TASK‐1 and the two TASK‐1 variants (see Methods).

Electrophysiological recordings showed that neither the GFP tag (9.7 pA pF^–1^, *n* = 9 cells, 95% CI = 6.8–12.7 for WT_TASK‐1 channels *vs*. 8.2 pA pF^–1^, *n* = 8 cells, 95% CI = 4.9–11.5 for pACGFP_TASK‐1 channels), nor the different transfection agent (8.0 pA pF^–1^, *n* = 5 cells, 95% CI = 5.8–10.1 for CaCl_2_ transfection *vs*. 9.5 pA pF^–1^, *n* = 5 cells, 95% CI = 8.3–10.7 for turbofect transfection), altered the magnitude of functional current through WT‐TASK‐1 channels, as measured in temporally matched experiments.

**Figure 4:**
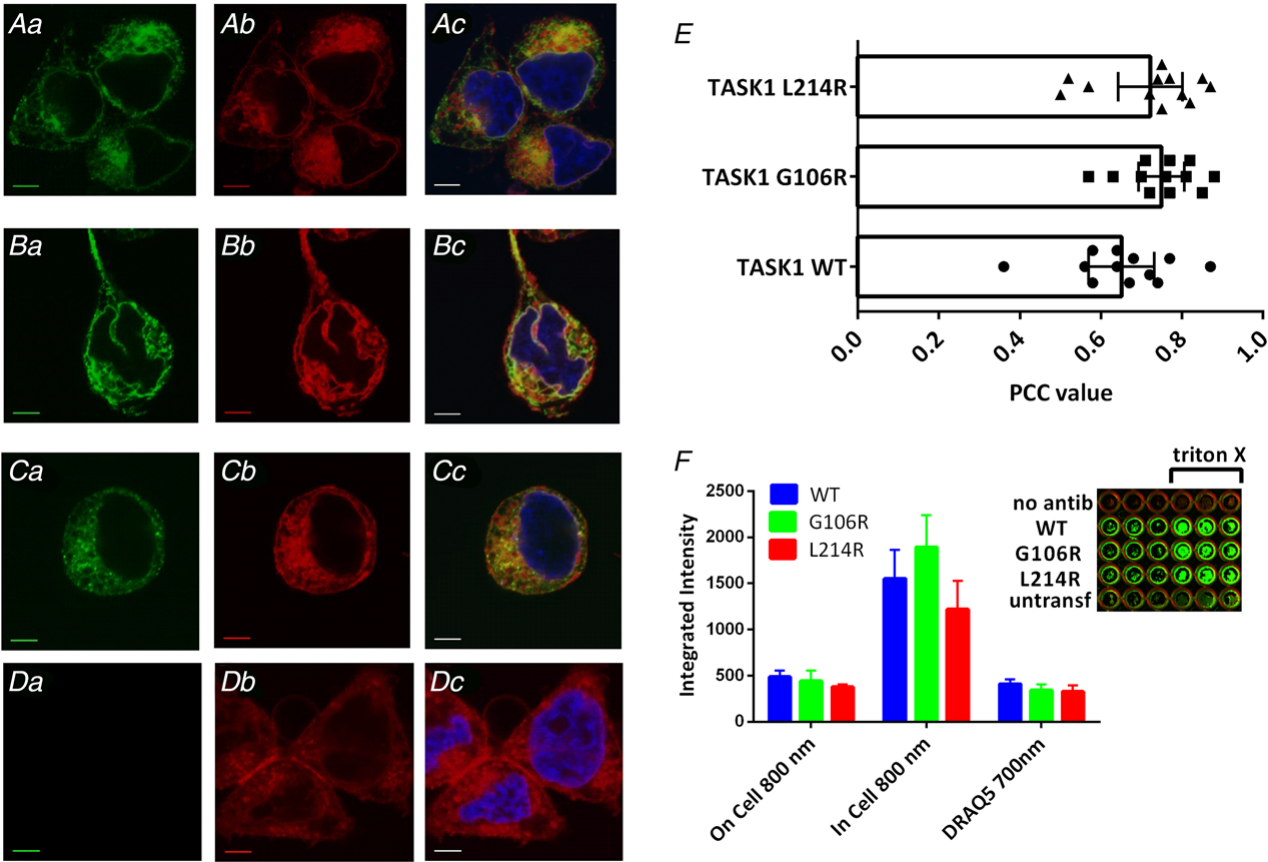
Evaluation of cellular localization of labelled TASK‐1 variants *Aa* and *Ab*, photomicrograph taken using confocal microscopy showing cellular localization of WT TASK‐1 channels C terminally fused with GFP, relative to the location of the plasma membrane stained with CellMask Deep Red. *Ac*, overlay of (*Aa*) and (*Ab*) indicating co‐localization of TASK‐1‐GFP with the plasma membrane in yellow. Nuclei were stained with the blue fluorescent dye Hoechst 33258. *Ba*, cellular localization of TASK‐1_G106R fused with GFP, relative to the location of the membrane (*Bb*). *Bc*, co‐localization of the G106R variant at the membrane in yellow. *Ca*, localization of TASK‐1_L214R in relation to the plasma membrane (*Cb*). *Cc*, overlap of the GFP‐fused variant with the plasma membrane, stained red. *Da*, cells untransfected with TASK‐1; (*Db*) and (*Dc*) as above. All scale bars are 5 μm. *E*, quantification of the co‐localization observed in experiments, as shown in (*Ac*), (*Bc*) and (*Cc*), using Pearson's correlation coefficient. A correlation coefficient of 1 represents 100% correlation. *F*, integrated fluorescence intensity for HA‐tagged WT and mutant TASK‐1 channels in non‐permeabilized (membrane) and permeabilized (whole‐cell) cells detected at 800 nm and for DRAQ5 (whole cell) detected at 700 nm using a Li‐Cor Odyssey SA fluorescence imager. Each column represents the mean ± SEM from three independent experiments performed in triplicate. The mean integrated intensity obtained from untransfected cells on the same plate, treated in the same way, was subtracted from each value. Inset: exemplar coverslips from a single plate, for ‘no antib’ (WT TASK‐1 with no primary antibody), WT TASK‐1, TASK‐1_G106R, TASK‐1_L214 transfected cells and untransfected cells ‘untransf’. Each row has three unpermeabilized coverslips and three permeabilized (with Triton X‐100) coverslips. [Color figure can be viewed at wileyonlinelibrary.com]

Cellular localization of WT TASK‐1‐GFP in tsA201 cells was examined using confocal microscopy. GFP fluorescence was observed at the plasma membrane of cells transiently transfected with WT TASK‐1‐GFP but there was also expression of WT TASK‐1 intracellularly. Figure 4 *Aa* represents a typical cell expressing TASK‐1‐GFP fused channels (green), excited at 480 nm. Fluorescence was also observed for the plasma membrane specific stain, CellMask Deep Red. Figure 4 *Ab* shows the fluorescence signal (red) for the same cell excited at 561 nm. Expression of the channel at the membrane was confirmed by co‐localization of the green signal of the channel with the red signal emitted from the plasma membrane specific stain (Fig. 4 *Ac*). The nuclei are stained blue. Co‐localization of WT TASK‐1 with the plasma membrane was quantified using PCC from 12 cells, from eight different plates from four different cultures. A strong linear correlation of 0.65 (*n* = 12 cells, 95% CI = 0.57–0.73) was observed for WT TASK‐1 at the membrane (Fig. 4 *E*). The same analysis for the two TASK‐1 variants gave results similar to that seen for WT TASK‐1 channels. Strong green fluorescence was observed at the membrane of cells transiently transfected with a GFP fused TASK‐1_G106R or TASK‐1_L214R mutant channels (Fig. 4 *Ba* and *Ca*), which showed some overlap with the red fluorescence from the membrane specific stain (Fig. 4 *Bb*, *Bc*, *Cb* and *Cc*). PCC values of 0.75 (*n* = 12 cells, 95% CI = 0.69–0.81 and 0.72 (*n* = 12 cells, 95% CI = 0.64–0.80 were calculated for TASK‐1_G106R and TASK‐1_L214R, respectively (Fig. 4 *E*), which was not significantly different from WT TASK‐1 (*P* > 0.05, one‐way ANOVA) suggesting that the two variants were both translated and trafficked to the membrane to the same degree as WT TASK‐1 under these experimental conditions. In comparison, untransfected cells showed no green fluorescence (Fig. 4 *Da*–*Dc*).

### In‐cell and on‐cell westerns

To quantify the expression of WT TASK‐1 and mutated channel protein in the cells and at the membrane, we used in‐cell and on‐cell westerns. An HA tag was engineered between positions alanine (50) and arginine (51) in the extracellular loop between the first transmembrane domain and the first pore region (M1/P1 loop) of WT TASK‐1 channels and the two mutant TASK‐1 channels. Thus, the signal seen in unpermeabilized cells is proportional to the amount of channel expressed at the cell membrane, whereas the signal in cells permeabilized with Triton X‐100 is proportional to the total number of channels expressed in the cells. The number of cells in each coverslip was quantified using DRAQ5, a far‐red DNA stain. Figure 4 *F* shows that there was no significant difference between the total number cells in coverslips for WT and mutated channels (DRAQ5 700 nm), the total amount of TASK channel protein expressed in the cells (in‐cell 800 nm) nor the amount of channel expression at the cell membrane (on‐cell 800 nm) (*P* > 0.05, two‐way ANOVA). Consistent with previous work (Renigunta *et al*. 2006, Mathie *et al*. 2010), the data show that the majority of TASK‐1 channel expression is intracellular, although the distributions of WT and mutated channels are similar in transfected cells.

### Functional characterization of TASK‐1 variants under conditions of alkalosis

**Figure 5:**
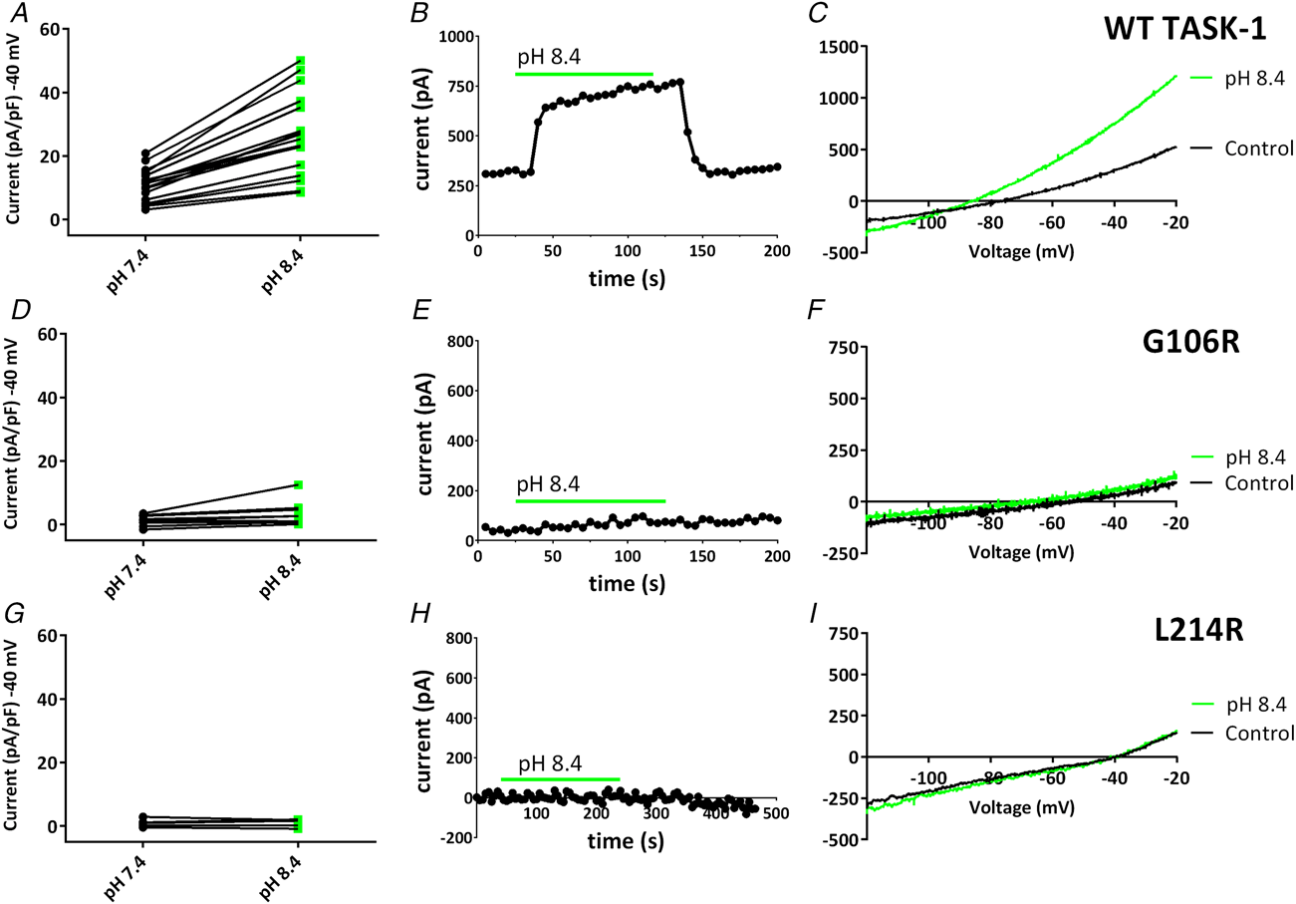
Effect of extracellular alkalosis on WT TASK‐1 and TASK‐1 variants *A*, plot of currents (pA pF^–1^) measured at –40 mV, recorded through WT TASK‐1 channels in either pH 7.4 (black dots) or pH 8.4 (green squares). A black line links the same cell in each condition. *B*, time course plot showing the effect of pH 8.4 on WT TASK‐1 currents. Each point is a 5 s average of the current at –40 mV. Application of pH 8.4 is indicated by the green bar. Current prior to and after the green bar is measured at pH 7.4. *C*, representative currents recorded through WT TASK‐1 in a single cell, evoked by ramp changes in voltage from –120 to –20 mV in pH 7.4 (black line) or pH 8.4 (green line). *D*–*F*, as shown for (*A*) to (*C*), but for TASK‐1_G106R channels. *G*–*I*, as shown for (*A*) to (*C*), but for TASK‐1_L214R channels. [Color figure can be viewed at wileyonlinelibrary.com]

The next step was to characterize the functionality of these variants using a variety of modulators and pharmacological tools. TASK‐1 channels have a p*K*_a_ of 7.3 and can be efficiently inhibited and activated by acidosis or alkalosis, respectively (Duprat *et al*. 1997). We investigated whether the TASK‐1 variants were still sensitive to pH changes, as well as whether it was possible to rescue functionality of these channels, by increasing extracellular pH from 7.4 to 8.4. As expected for WT TASK‐1 a change in pH_o_ from 7.4 to 8.4 caused a significant increase in outward current density measured at –40 mV (*P* < 0.05, paired *t* test, 95% CI of difference = 11.6–20.0) (Fig. 5 *A*–*C*). For the TASK‐1_G106R variant, there was no significant effect of external alkalosis (*P* > 0.05, paired *t* test, 95% CI of difference = –0.2 to 3.1]) (Fig. 5 *D*–*F*). Similarly, for TASK‐1_L214R, there was also no significant effect of external alkalosis (*P* > 0.05, paired *t* test, 95% CI of difference = –1.0 to 1.0]) (Fig. 5 *G*–*I*).

### Effect of ONO‐RS‐082 on novel TASK‐1 variants

**Figure 6:**
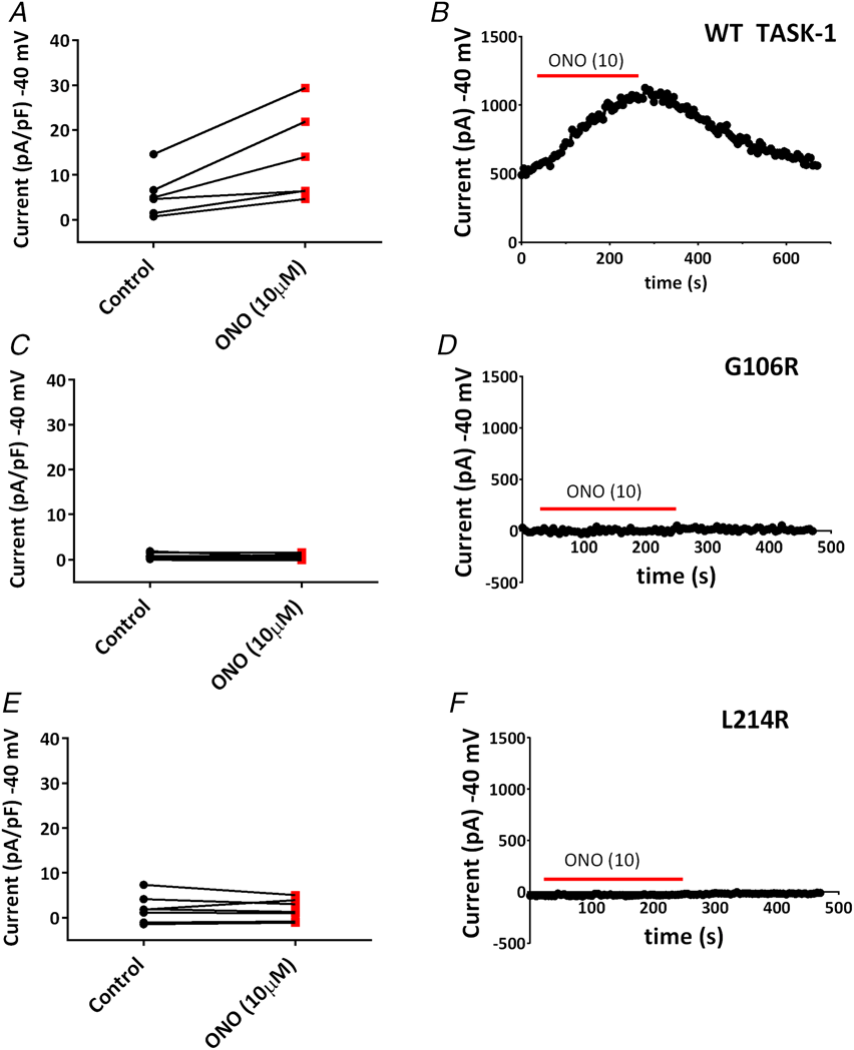
Effect of ONO‐RS‐082 (10 μM) on WT TASK‐1 and TASK‐1 variants *A*, a plot of currents (pA pF^–1^) measured at –40 mV, recorded through WT TASK‐1 channels in either extracellular recording solution (control) (black dots) or control solution containing 10 μm ONO‐RS‐082 (red squares). A black line links the same cell in each condition. *B*, time course plot showing the acute application of ONO‐RS‐082 (10 μm) on WT TASK‐1 currents. Each point is a 5 s average of the current at –40 mV. Application of ONO‐RS‐082 is indicated by the red bar. Current prior to and after the red bar is measured in control solution. *C* and *D*, as shown for (*A*) and (*B*), but for TASK‐1_G106R channels. *E* and *F*, as shown for (*A*) and (*B*), but for TASK‐1_L214R channels. [Color figure can be viewed at wileyonlinelibrary.com]

It has previously been reported that the phospholipase A2 inhibitor, ONO‐RS‐082, was able to rescue current through two of six heterozygous TASK‐1 mutations identified in patients with PAH (Ma *et al*. 2013). As reported by Ma *et al*. 2013, acute application of 10 μm ONO‐RS‐082 to WT TASK‐1 resulted in a significant increase in current density (*P* < 0.05, paired *t* test, 95% CI of difference = 2.3–14.3) (Fig. 6 *A* and *B*). This effect could be reversed in wash (Fig. 6 *B*). By contrast, current density through the two mutant TASK‐1 channels could not be recovered by ONO‐RS‐082 using the same experimental conditions. For TASK‐1_G106R, *P* > 0.05, paired *t* test, 95% CI of difference = –0.7 to 0.5] and for TASK‐1_L214R, *P* > 0.05, paired *t* test, 95% CI of difference = –1.5 to 1.0] (Fig. 6 *C*–*F*).

### Riociguat‐induced enhancement of TASK‐1 current

**Figure 7:**
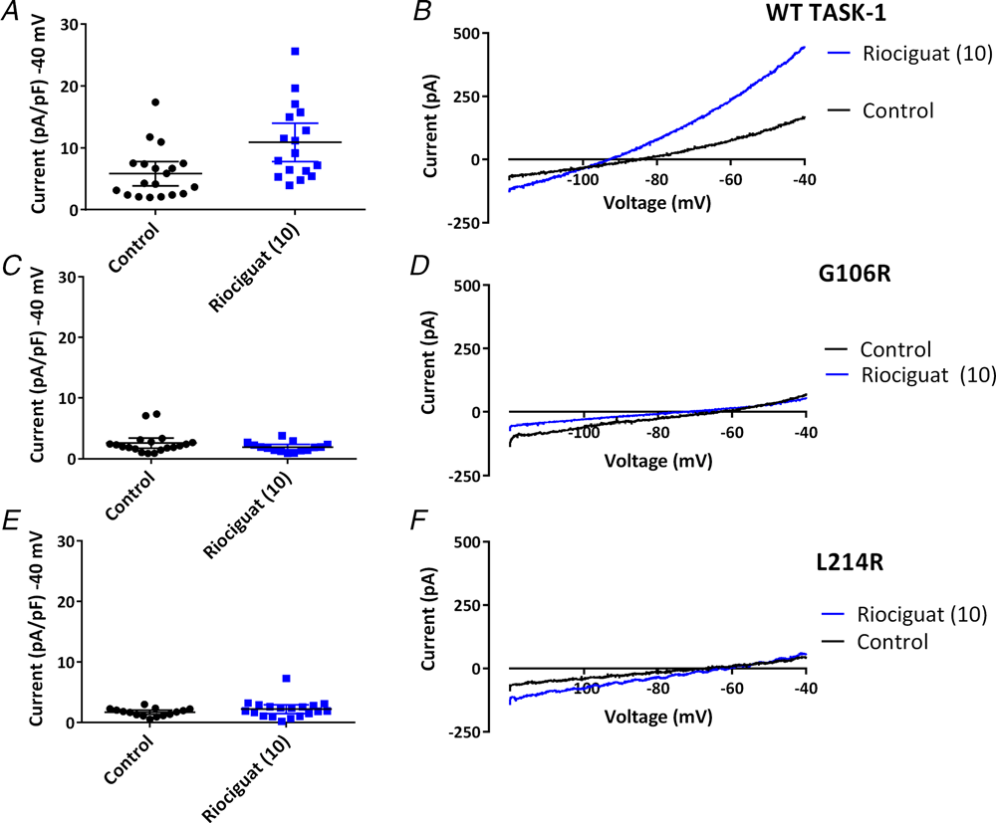
Effect of Riociguat (10 μm) on WT TASK‐1 and TASK‐1 variants *A*, plot of current (pA pF^–1^) measured at –40 mV from individual cells transiently expressing WT TASK‐1 either incubated in extracellular solution minus riociguat (control: black dots) or incubated in extracellular solution containing 10 μm riociguat (blue squares). Error bars represent the 95% CI. *B*, representative currents recorded through WT TASK‐1, evoked by ramp changes in voltage from –120 mV to –40 mV under control conditions (2.5 mm [K^+^]_o_), (black line) and after incubation in 10 μm riociguat (blue line). *C* and *D*, as shown for (*A*) and (*B*), but for TASK‐1_G106R channels. *E* and *F*, as shown for (*A*) and (*B*), but for TASK‐1_L214R channels. [Color figure can be viewed at wileyonlinelibrary.com]

A relatively recent drug licensed for the treatment of PAH, riociguat belongs to a novel class of pharmacological agents that directly stimulate soluble guanylate cyclase (sGC), a key enzyme in the nitric oxide–cGMP signalling pathway, a major player in the pathophysiology of PAH. Riociguat stimulates the production of cGMP, which acts to regulate vascular tone by controlling dilatation and cellular proliferation of the vascular wall. We aimed to investigate the action of the sGC stimulator, riociguat, on WT TASK‐1 and the TASK‐1 variants. Incubation of WT TASK‐1 channels in either extracellular solution containing 10 μm riociguat or extracellular solution minus drug for 20 min before recording from cells resulted in a significant increase in current measured at –40 mV (*P* < 0.05, unpaired *t* test, 95% CI of difference = 1.6–8.5) from 5.8 pA pF^–1^ (*n* = 19, 5 days, 95% CI = 3.8–7.8) for control cells to 10.9 pA pF^–1^ (*n* = 17, 5 days, 95% CI = 7.8–14.0) for cells incubated in riociguat (Fig. 7 *A* and *B*). The increased current through TASK‐1 channels in riociguat led to a significant hyperpolarization of the zero current level (*V*_M_) (*P* < 0.05, unpaired *t* test, 95% CI of difference = 7.8–23.0) from –77 mV (95% CI = –72 to –82) to –93 mV (95% CI = –87 to –99).

Using the same experimental procedure as described for WT TASK‐1, we investigated the effect of riociguat (10 μm) on the two TASK‐1 variants aiming to determine whether current could be rescued through these mutant channels. As seen for ONO‐RS‐082 and pH 8.4, no increase in functional current by riociguat was observed for either TASK‐1_G106R or TASK‐1_L214R. Incubation of TASK‐1_G106R channels in either extracellular solution containing 10 μm riociguat or extracellular solution minus drug for 20 min before recording from cells resulted in no significant difference in current measured at –40 mV (*P* > 0.05, unpaired *t* test, 95% CI of difference = –1.7 to 0.3) (Fig. 7 *C* and *D*). Similarly, incubation of TASK‐1_L214R channels in either extracellular solution containing 10 μm riociguat or extracellular solution minus drug for 20 min before recording from cells resulted in no significant difference in current measured at –40 mV (*P* > 0.05, unpaired *t* test, 95% CI of difference = –0.3 to 1.4) (Fig. 7 *E* and *F*). By contrast to cells expressing WT channels, riociguat had no significant effect on the zero current level (*V*_M_) of cells expressing TASK‐1_G106R channels (*P* > 0.05, 95% CI of difference = –12.6 to 5.7) or TASK‐1_L214R channels (*P* > 0.05, 95% CI of difference = –9.8 to 8.5).

## Discussion

In the present study, we report for the first time the functional consequences of two mutations recently reported in PAH patients. We show that current through two new homozygous mutations (G106R, L214R) of TASK‐1 channels is considerably reduced compared to WT TASK‐1 channels. Although the two mutations of TASK‐1 considerably reduce channel current, this is not the result of any trafficking defects because WT and mutant channels appear to be equally distributed at the cell membrane. Instead, the two mutations must alter the functional properties of TASK‐1 channels to reduce current. In both cases, G106R and L214R, two large positively‐charged arginine residues, are substituted into the channel, close to the selectivity filter (Fig. 1), which might be hypothesized to interfere with flow of potassium ions through the channel.

TASK‐1 is expressed in rabbit, mouse, human and rat PASMCs (Gurney *et al*. 2003, Gardener *et al*. 2004, Olschewski *et al*. 2006, Manoury *et al*. 2009). The non‐inactivating K current in rat, rabbit and human PASMCs has functional characteristics of TASK‐1 channels (Olschewski *et al*. 2017), although this may not be the case in mouse PASMCs (Manoury *et al*. 2009, 2011). TASK‐1 channels are important regulators of PASMC resting membrane potential and excitability. In rat models of PAH, TASK‐1 current decreased progressively during development of the disease and this was associated with a membrane depolarization (Antigny *et al*. 2016). Indeed, the results of the present study suggest that reduced function of TASK‐1 channel activity is important in both hPAH and iPAH.

Pharmacological enhancement of WT TASK‐1 current is observed with both alkaline pH (8.4) and application of the phospholipase A2 inhibitor, ONO‐RS‐082. In rat models of PAH, treatment with ONO‐RS‐082 reversed the proliferation, vasoconstriction and inflammation that was observed (Antigny *et al*. 2016), indicating the importance of TASK‐1 channels in PAH.

Importantly, in some of the TASK‐1 mutations (T8K, E182K), but not all (e.g. G203D) described by Ma *et al*. (2013), ONO‐RS‐082 was able to restore current through mutant channels. However, this only occurred at membrane potentials more positive to the resting membrane potential of the cells and more positive to that seen for enhancement of WT TASK‐1 current. Similarly, we have shown previously that current through the mutated TASK‐3 channel (G236R) that underlies KCNK9 imprinting syndrome (Graham *et al*. 2016) can be recovered both pharmacologically through application of fenamate compounds and genetically through further gain of function channel mutations (Veale *et al*. 2014). These pharmacological interventions restore both current amplitude and the apparent K selectivity in the channel (Veale *et al*. 2014; Bohnen *et al*. 2017) because, without the latter, enhancement of a current with reduced K selectivity would exacerbate any pathophysiological responses resulting from cellular depolarization, as would be the case for PASMCs in PAH (Olschewski *et al*. 2017). However, neither of the two mutant channels in the present study showed any recovery of current following exposure to either alkaline pH or ONO‐RS‐082.

Although the two mutations in the present study have been observed as homozygous mutations in patients, previously identified mutations were heterozygous and heterozygous channel dimers did display functional TASK‐1 current, although this was considerably reduced compared to WT TASK‐1 (Ma *et al*. 2013; Bohnen *et al*. 2017). Current through these heterozygous mutations could be restored by ONO‐RS‐082 (Bohnen *et al*. 2017). For the two mutations in the present study, current through heterozygous channels was no larger than that seen for the homozygous mutants. Furthermore, mutant TASK‐1 channels (such as WT TASK‐1 channels) (Czirjak & Enyedi 2002, Berg *et al*. 2004, Kang *et al*. 2004) could form functional heteromeric channels with TASK‐3 channels (Bohnen *et al*. 2017). Homomeric TASK‐1 channels are the predominant TASK channels in PASMCs, particularly human PASMCs (Olschewski *et al*. 2006), and the role of these channels in regulating pulmonary vascular tone and controlling the resting membrane potential of these cells is well established in humans (Boucherat *et al*. 2015). However, in other tissues, such as atrial cardiomyoctes (Rinne *et al*. 2015), carotid body glomus cells (Kim *et al*. 2009) and cerebellar granule neurons (Aller *et al*. 2005), the effect of TASK‐1 channel mutations could be somewhat mitigated through the formation of functional heterodimers with TASK‐3 channel subunits in these cells.

The guanylate cyclase activator, riociguat, is approved for the treatment of PAH. Riociguat catalyses the synthesis of cGMP, and activates protein kinase G (PKG), which acts through several putative mechanisms to reduce intracellular Ca and inhibit smooth muscle contraction (Ghofrani *et al*. 2017).

Previous studies have shown that cGMP can enhance current through both recombinant TASK‐1 channels in expression systems and native TASK‐1 currents in basal forebrain neurons (Kang *et al*. 2007, Toyoda *et al*. 2008, 2010). Similarly, TNFα activation of TASK‐3 channels (El Hachmane *et al*. 2014) is considered to be mediated through cGMP. It has been proposed that cGMP activates PKG, which then tunes the pH sensitivity of TASK‐1 channels, through PKG‐mediated phosphorylation, such that the phosphorylated channels are less inhibited by H ions at physiological pH values (Toyoda *et al*. 2010).

In the present study, we show for the first time that riociguat enhances current through WT TASK‐1 channels, which could contribute to its therapeutic benefit in PAH. However, riociguat does not have any effect on current through G106R or L214R mutated TASK‐1 channels. Whether this is important in terms of the therapeutic strategy for patients with these mutations will depend on whether the primary action of riociguat in PAH is mediated through activation of TASK‐1 channels or whether this is an additional (but beneficial) action of riociguat (Ghofrani *et al*. 2017). In this regard, it is of interest that other regulatory pathways contributing to cell proliferation and vasoconstriction in the pulmonary arteries alter the function of TASK‐1 channels in PASMCs. For example, endothelin‐1, a potent vasoconstrictor that stimulates vascular remodelling, inhibits TASK‐1 channels via both a protein kinase C‐dependent pathway (Tang *et al*. 2009, Schiekel *et al*. 2013) and Rho kinase (Seyler *et al*. 2012). Similarly, treprostinil, a stable analogue of prostacyclin, acts via PKA to up‐regulate TASK‐1 current in human PASMCs (Olschewski *et al*. 2006).

Down‐regulation or inhibition of TASK‐1 channel activity has been proposed to contribute to vascular remodelling in PAH (Olschewski *et al*. 2006, Tang *et al*. 2009, Antigny *et al*. 2016) and a loss of TASK‐1 channel function and expression is a characteristic of right ventricular hypertrophy associated with pulmonary hypertension (Lambert *et al*. 2018). Guanylate cyclase activators, endothelin receptor antagonists and prostacyclin analogues are established vascular therapeutic strategies in the treatment of PAH, acting to reduce vasoconstriction and smooth muscle proliferation, and all three will stimulate activation of TASK‐1 channels in PASMCs, which would contribute to their therapeutic benefit.

In summary, novel mutations in TASK‐1 channels recently seen in PAH are associated with a loss of function. Current through these channels could not be restored by activators of TASK‐1 channels. Riociguat enhancement of current through TASK‐1 channels could contribute to its therapeutic benefit in the treatment of PAH.

## Additional information

### Competing interests

The authors declare that they have no competing interests.

### Author contributions

ALC, AM, PE‐S and ELV participated in the research design. KPC, RGH and ELV conducted the experiments. KPC, RGH, ELV and AM performed the data analysis. AM and ELV wrote the manuscript. KPC, RGH, ALC, PE‐S, ELV and AM revised the final manuscript and approved the version submitted for publication.

### Funding

This study was supported by the Biotechnology and Biological Sciences Research Council (UK) [BB/J000930/1] (AM, ELV); the Spanish Ministerio de Economia y Competitividad (SAF2016‐77222‐R to AC) with funds co‐financed by ERDF (FEDER) Funds from the European Commission, ‘A way of making Europe’; by Comunidad de Madrid en Biomedicina (B2017/BMD‐3727) (AC); and by the Cardiovascular Medical Research and Education Fund (USA) (AC, AM).
